# Influence of 3d Transition Metal Doping on Lithium Stabilized Na-β″-Alumina Solid Electrolytes

**DOI:** 10.3390/ma14185389

**Published:** 2021-09-17

**Authors:** Cornelius L. Dirksen, Karl Skadell, Matthias Schulz, Micha P. Fertig, Michael Stelter

**Affiliations:** Fraunhofer Institute for Ceramic Technologies and Systems IKTS, Michael-Faraday-Str. 1, 07629 Hermsdorf, Germany; Karl.Skadell@ikts.fraunhofer.de (K.S.); Matthias.Schulz@ikts.fraunhofer.de (M.S.); Micha.Philip.Fertig@ikts.fraunhofer.de (M.P.F.); Michael.Stelter@ikts.fraunhofer.de (M.S.)

**Keywords:** doping, Na-β″-alumina, sodium−ion battery, sodium−ion conductor, solid electrolyte

## Abstract

Na-β″-alumina is the commercially most successful solid electrolyte due to its application in ZEBRA and NAS^®^ batteries. In this work, Li-stabilized Na-β″-alumina electrolytes were doped with 3d transition metal oxides, namely TiO_2_, Mn_3_O_4_, and NiO, in order to improve their ionic conductivity and fracture strength. Due to XRD and EDX measurements, it was concluded that Mn- and Ni-ions are incorporated into the crystal lattice of Na-β″-alumina. In contrast, TiO_2_ doping results in the formation of secondary phases that enable liquid-assisted sintering at temperatures as low as 1500 °C. All dopants increased the characteristic fracture strength of the electrolytes; 1.5 wt% of NiO doping proved to be most efficient and led to a maximal characteristic fracture strength of 296 MPa. Regarding the ionic conductivity, TiO_2_ doping showed the uppermost value of up to 0.30 S cm^−1^ at 300 °C. In contrast to the other dopants, TiO_2_ doping lowered the sintering temperature needed to obtain a dense, stable, and highly conductive Na-β″-alumina electrolyte suitable for applications in Na based batteries.

## 1. Introduction

Due to their high conductivity for Na-ions compared to other solid electrolytes [1], polycrystalline electrolytes made from Na-β″-alumina are established in commercial Na-batteries like ZEBRA or NAS^®^ batteries since the 1990s [2]. The excellent conductivity for Na-ions is caused by highly conductive crystallographic planes occupied by Na-ions. The conduction planes are separated by two densely packed nonconductive spinel blocks, which results in a significant anisotropic conductivity within the crystallites. Accordingly, a high overall conductivity in a polycrystalline electrolyte body requires the presence of crystallites that provide a statistically distributed spatial orientation. In consequence, the electrolyte microstructure is of tremendous importance for material quality. Doping of Na-β″-alumina with 3d transition metals is known to influence the microstructure and is an excellent material scientific approach to optimize the electrolyte performance, and manufacturing processes them off. In the present paper, results from systematic doping experiments are reported, and fundamental mechanisms and correlations are derived.

The research on Na-β″-alumina electrolytes has so far mainly focused on different production techniques [3,4,5,6,7,8] or the sintering behavior [9,10,11]. Some attention is also paid to improve the ionic conductivity and fracture strength of Na-β″-alumina electrolytes by doping.

The high ionic conductivity of the Na-β″-alumina electrolyte lowers the overall resistance of an electrochemical cell and increases, therefore, its efficiency. The fracture strength is important because Na-β″-alumina electrolytes have to withstand temperature and pressure variations within a cell [12]. Furthermore, the necessary wall thickness of the electrolyte, and thereby the resistance, can be lowered if the characteristic fracture strength of the material is enlarged. Consequently, 3d transition metal doping of Na-β″-alumina can help to improve the electrochemical cell characteristics of sodium-based battery systems and strengthen their economic position.

Na-β″-alumina is typically doped with Mg^2+^ or Li^+^ to prevent decomposition of Na-β″-alumina at the commonly applied sintering temperatures of about 1600 °C [8,13,14]. Typical compositions for stabilized Na-β″-alumina electrolytes are Na_1.67_Mg_0.67_Al_10.33_O_17_ or Na_1.67_Li_0.33_Al_10.67_O_17_ [13,15,16].

Additionally, up to 15 vol.% ZrO_2_ is a common dopant that inhibits extensive grain growth of Na-β″-alumina grains by forming intergranular ZrO_2_ particles. However, the addition of isolating ZrO_2_ is known to lower the ionic conductivity of Na-β″-alumina electrolytes [8,17,18].

3d transition metal doping has shown a high potential to promote ionic conductivity and characteristic fracture strength [13,19,20,21,22]. Nevertheless, the measured conductivities are difficult to compare because different reactants for synthesis, co-dopants, synthesis routes, and measuring temperatures were used from various researches [23,24,25,26]. For this reason, three promising 3d metal dopants for Na-β″-alumina, namely TiO_2_, Mn_3_O_4_, and NiO, are of interest in this work.

Compared to other 3d dopants, Ti doping was under investigation in literature repeatedly [13,21,23,24]. It has shown the most beneficial influence on the ionic conductivity of Na-β″-alumina. The reasons for this large improvement compared to other dopants is not clear yet. Lu et al. [27] proposed but did not observe the formation of a transient liquid phase containing compounds such as Na_2_Ti_3_O_7_, Na_8_Ti_5_O_14_, or Na_2_Ti_6_O_13_. The liquid phase could form at temperatures of about 1300 °C and promote the densification process. Furthermore, an increased number of Al^3+^ vacancies could promote diffusion and thereby improve the sintering behavior. This mechanism is widely accepted for the increased densification of TiO_2_-doped Al_2_O_3_ [28,29].

This process also might be possible for Mn- or Ni-doped Na-β″-alumina.

A comprehensive evaluation of Mn-doped Na-β″-alumina has not been published so far. Kennedy et al. [25] doped Mg^2+^ stabilized Na-β″-alumina with 1, 2, and 4 wt% Mn(NO_3_)_2_ and found an increase of the ionic conductivity from 0.018 to 0.025 S cm^−1^ at 300 °C for a doping amount of 4 wt%. The characteristic fracture strength and the influence of different sintering regimes were not analyzed. Lu et al. [27] tested co-doped Na-β″-alumina (5 wt% Mn(NO_3_)_2_, Ti(OCH(CH_3_)_2_)_4_, and ZrO_2_). The ionic conductivity or the characteristic fracture strength of Mn(NO_3_)_2_-single-doped Na-β″-alumina was not reported, but Lu et al. noted a slightly increased shrinkage while sintering Mn(NO_3_)_2_-doped Na-β″-alumina.

Zhu et al. [20] and Kennedy et al. systematically investigated Ni-doped Na-β″-alumina [25]. Zhu et al. found a conductivity maximum by doping with 0.25 wt% NiO (0.066 S cm^−1^ at 350 °C), while Kennedy et al. found a steady increase of the conductivity up to the maximum tested doping amount of 4 wt% Ni(NO_3_)_2_ (0.047 S cm^−1^ at 300 °C). Both reported ionic conductivities are in comparison to undoped Na-β″-alumina in other publications well below the average [4,30]. This indicates a poor microstructure, a low relative density, a low phase content, or other barriers. Moreover, Zhu et al. reported a bending strength, which increased from 194 to 296 MPa after the addition of 0.25 wt% NiO [20].

Here, we close the lack of comparable and comprehensive data regarding 3d transition metal doping of Na-β″-alumina. Therefore, this work cites and extends some results of a former publication [13] of the authors about TiO_2_ doping. Furthermore, it investigates the key characteristics, such as conductivity and characteristic fracture strength of Mn_3_O_4_ and NiO-doped Na-β″-alumina electrolytes at different doping amounts and sintering temperatures. In contrast to previous studies, various dopants, doping amounts, and sintering temperatures were tested. Density, SEM–EDX, and XRD studies were carried out to further investigate the so far poorly addressed mechanisms of 3d transition metal doping of Na-β″-alumina.

## 2. Materials and Methods

### 2.1. Sample Preparation

AlO(OH) (>98%; Nabaltec, Schwandorf, Germany), Na_2_CO_3_ (>99%; Carl Roth, Karlsruhe, Germany), and Li_2_CO_3_ (>99.8%; Carl Roth, Karlsruhe, Germany) were mixed in the stoichiometry Na_1.67_Al_10.67_Li_0.33_O_17_. Afterwards, the powder was calcined at 1280 °C / 2 h in MgO crucibles. The obtained white powder was mixed with TiO_2_ (>99.7%; Alfa Aesar, Haverhill, MA, USA) or NiO (>99%; Lomberg Chemie, Oberhausen, Germany) without any further treatment. MnO_2_ (>98%; Carl Roth, Karlsruhe, Germany) was oxidized to Mn_3_O_4_ (650 °C/5 h) before using it as a dopant. The powder mixture was then suspended in EtOH and ball milled in ZrO_2_ beakers for 0.5 h. After drying (80 °C/12 h), granulating (with an organic binder), and pressing (up to 110 kN), the ceramic green bodies were sintered under air atmosphere at 1500 °C, 1600 °C, or 1700 °C for 0.5 h. This resulted in transition-metal-doped solid electrolyte bodies. To calculate the molar ratio presented in Section 3, the amount of added transition metal ions was divided by the amount of Na-β″-alumina with the stoichiometry Na_1.67_Al_10.67_Li_0.33_O_17_. The mass fraction of the dopants is always given in percentage.

### 2.2. Sample Characterization

XRD patterns were measured from grounded disks (D8 Advance, Bruker, Billerica, MA, USA). The quantitative XRD evaluation was performed by the Rietveld refinement method (Autoquan 2.8.0.2).

Density analysis was carried out via Archimedes’ principle in toluene.

The absolute density was measured by a He-Pycnometer (Pyknomatik-ATC, Thermo Fisher, Waltham, MA, USA).

To take SEM images (Ultra 55+, Carl Zeiss, Oberkochen, Germany), fractured disks were used. SEM–EDX-scans (Ultra 55+, Carl Zeiss, Oberkochen, Germany/Trident XM4, EDAX, Mahwah, NJ, USA) were taken from polished disks.

The fracture strength was tested on ten sintered tablets per doping level by ball-on-three-balls method (Zwick 100, Zwick, Ulm, Germany). The data were evaluated by Weibull statistics (maximum likelihood estimation).

**Figure 1:**
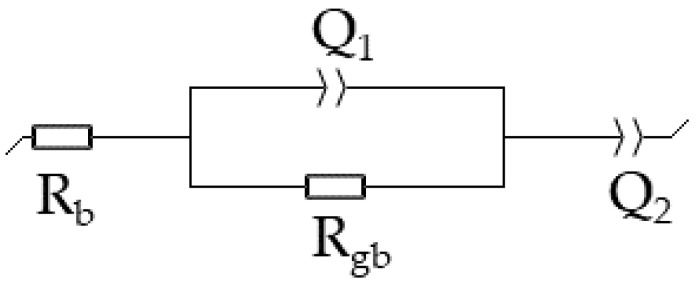
Equivalent circuit used for fitting the Nyquist plot. R_b_ represents the starting point of the semicircle at high frequencies. R_gb_ and the constant phase element Q_1_ represent the size of the semicircle. Q_2_ represents the low-frequency area caused by electric contact and is of no further interest for this work.

Ionic conductivities were measured from bar-shaped samples. The measurements were carried out at two separately sintered bars of each sample by impedance spectroscopy (SP-240, Biologic, Seyssinet-Pariset, France and Reference 3000 AE, Gamry Instruments, Warminister, PA, USA) at a temperature of 300 °C. The range of the applied frequencies was from 1 MHz to 9 Hz. The sinus amplitude was set to 10 mV. Figure 1 displays the equivalent circuit, which was used to fit the Nyquist plots. The bulk- (R_b_) and the grain boundary resistance (R_gb_) were selected from the regression analysis curve to calculate the resistance (R) of the sample Equation (1).

(1) $$R=R_{b}+R_{gb}$$

To calculate the ionic conductivity (σ), Equation (2) was used. L represents the length of the sample and A, the cross-sectional area.

(2) $$\sigma =\frac{L}{A\cdot R}$$

The calculation of the specific grain boundary resistance (R_sgb_) was done by Equation (3).

(3) $$R_{sgb}=\frac{A\cdot R_{gb}}{L}$$

The measurements were realized in the sample-holders shown in a previous publication [13].

## 3. Results

### 3.1. XRD Analysis

**Figure 2:**
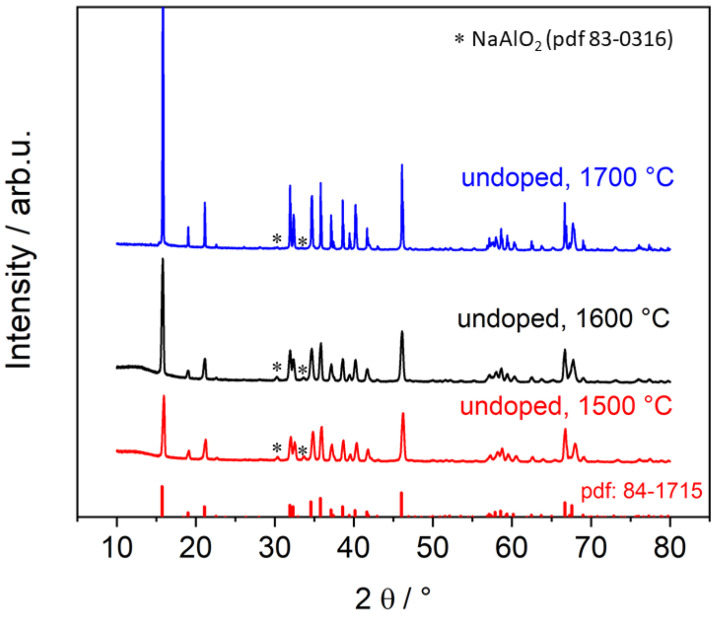
Diffraction patterns of undoped Na-β″-alumina sintered at 1500 °C, 1600 °C, or 1700 °C. The literature reflexes of Na-β″-alumina (pdf 84-1715) are illustrated by the red bars at the bottom. The reflex 006 (15.8°) is clipped off.

To verify the phase composition and detect any secondary phases of the Na-β″-alumina electrolytes, XRDs were recorded. Firstly, the undoped reference materials after sintering at temperatures of 1500 °C, 1600 °C, and 1700 °C were measured as can be seen in Figure 2. The diffraction patterns are in good agreement with the literature-reported reflexes of Na-β″-alumina. The reflexes at 30.2° and 33.6° can be addressed to NaAlO_2_ impurities. Rietveld refinement gave a Na-β″-alumina phase content of >95% for all three samples. The exact results are displayed in Table 1. As a difference in between the samples, the reflex 006 at 15.8° leaps to the eye. The reflex shows an increasing intensity as the sintering temperature rises. Li et al. [31] addressed this phenomenon to increasing anisotropic grain growth.

#### 3.1.1. Phase Composition of TiO_2_-Doped Electrolytes

**Figure 3:**
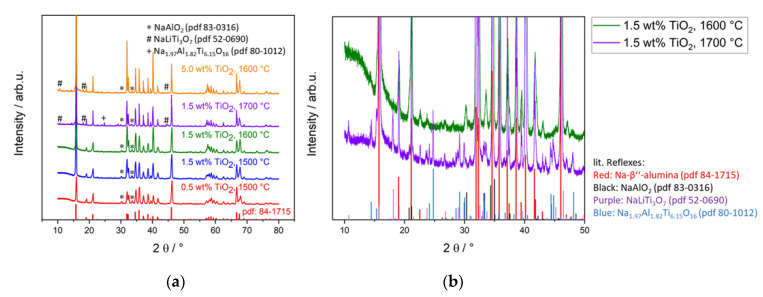
(**a**) Diffraction patterns of TiO_2_-doped Na-β″-alumina samples. The reflex 006 (15.9°) is clipped off. The literature reflexes at the bottom are taken from pdf 84-1715. (**b**) XRD fingerprint of two TiO_2_-doped Na-β″-alumina samples with Na-β″-alumina literature reflexes at the bottom.

XRD patterns of TiO_2_-doped Na-β″-alumina, displayed in Figure 3, also show a high agreement with literature-reported reflexes. The Na-β″-alumina phase content of all the TiO_2_-doped samples is constantly about 95%. Neither doping amount nor sintering temperature has a large influence, as displayed in Table 1.

Diffraction patterns of samples with lower TiO_2_ doping amounts (≤2.5 wt%) and lower sintering temperatures (≤1600 °C) do not indicate the formation of any secondary phases besides Na-β″-alumina, NaAlO_2_, and some negligible amounts of Na-β-alumina. XRD patterns of highly doped samples and/or samples sintered at 1700 °C clearly show the formation of two additional phases next to NaAlO_2_ and Na-β″-alumina, namely NaLiTi_3_O_7_ and Na_1.97_Al_1.82_Ti_6.15_O_16_. Those secondary phases might also be present in samples from sintering at lower temperatures, but they are not detectible by XRD due to a lower crystalline fraction of small crystallites. The latter phenomenon would lead to spread out reflexes, which cannot be detected under the noise (compare XRD amorphousness). Figure 3b exemplarily presents the fingerprint areas of a sample doped with 1.5 wt% TiO_2_ and a sintering temperature of 1700 °C, with clearly visible secondary phases beside NaAlO_2_. Additionally, the same section of an XRD pattern taken from a sample with the same doping amount but a sintering temperature of only 1600 °C is depicted. Here, no clear secondary phases besides NaAlO_2_ are detected.

Wei et al. [21] found an increase of the Na-β″-alumina phase content of 1.9% by doping with 1.75 wt% TiO_2_, while Yi et al. [18] reported a decrease for all tested doping amounts. Neither of those researchers nor other ones observed any Ti-containing secondary phase [21,23,27,32]. This is in line with the results in this work since samples doped as high as 5.0 wt% TiO_2_ or sintering temperatures of 1700 °C were not described in those publications.

**Figure 4:**
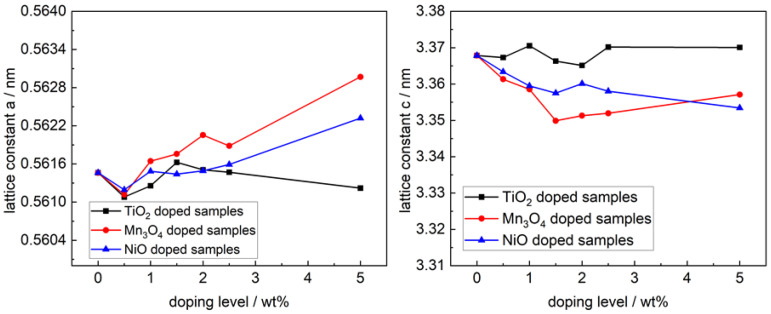
Crystallographic lattice parameters a and c of Na-β″-alumina samples sintered at 1600 °C.

The lattice parameters of the Na-β″-alumina phase were calculated for all doped samples sintered at 1600 °C from XRD data. In Figure 4, the results are given comparatively. The lattice parameters do not show a significant dependency between the amount of dopant and spread around the mean value of 0.5614 nm ± 0.0002 nm (parameter a, b) or 3.368 nm ± 0.002 nm (parameter c).

Boilot et al. [33] proposed that ions with radii smaller than 0.097 nm can substitute Al-ions in the Na-β″alumina lattice and stabilize the Na-β″-alumina phase. This is valid for all three tested dopants. However, the constant lattice parameters of TiO_2_-doped samples do not support the assumption that Ti-ions substitute Al-ions under the synthesis conditions tested in this work.

#### 3.1.2. Phase Composition of Mn_3_O_4_- and NiO-Doped Electrolytes

**Figure 5:**
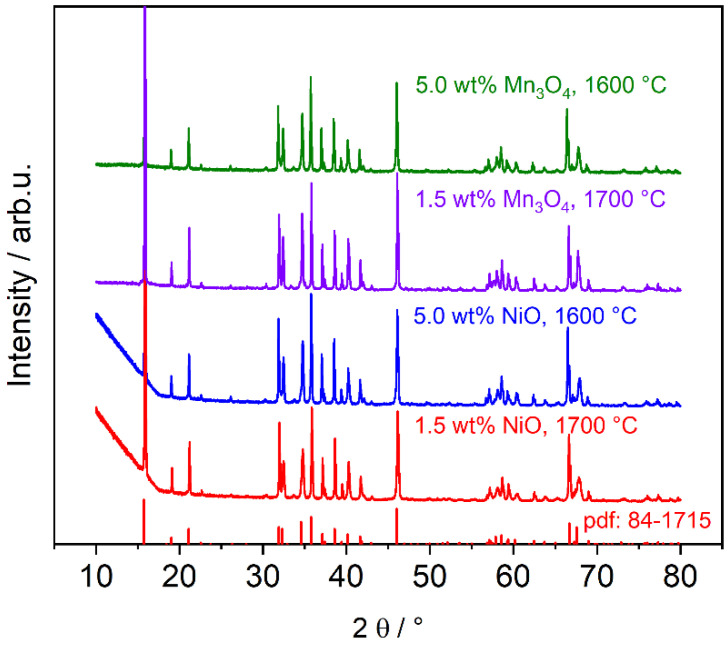
Diffraction patterns of Mn_3_O_4_- and NiO-doped Na-β″-alumina. The reflex 006 (15.9°) is clipped off. The literature reflexes of Na-β″-alumina (pdf 84-1715) are illustrated by the red bars at the bottom.

The XRD measurements of Mn_3_O_4_- and NiO-doped Na-β″-alumina electrolytes reveal no secondary phases besides NaAlO_2_ and minor amounts of Na-β-alumina. They also reveal a high agreement with the literature diffractogram of Na-β″-alumina, as Figure 5 exemplarily shows. The Na-β″-alumina phase contents listed in Table 1 indicate that Mn_3_O_4_- and NiO doping do not heavily affect the Na-β″-alumina phase content. Zhu et al. [20] found an increased Na-β″-alumina phase content from 92.3% to 98.9% by doping with 0.25 wt% NiO. In contrast to this work, Zhu. et al. used the intensity relation of a Na-β″-alumina reflex and a Na-β-alumina reflex to calculate the phase content. Phases such as Al_2_O_3_ or NaAlO_2_ were not taken into account.

The lattice parameters a and b of Mn_3_O_4_- and NiO-doped samples (Figure 4) increased slightly from 0.5615 nm to 0.5644 nm and 0.5618 nm due to a doping amount of 5.0 wt%. The lattice parameter c decreased at the same doping amount from 3.3679 nm to 3.3571 nm and 3.3536 nm, respectively. A change of the lattice constant, likely caused by different radii and electrostatic effects, hints at the Na-β″-alumina lattice incorporated Mn- and Ni-ions.

### 3.2. Density Analysis

A high relative density of the sintered Na-β″-alumina electrolytes is desirable because pores hinder the Na-ions passing through the electrolyte in the shortest possible way. Thereby, the ionic resistance of the electrolyte rises. Additionally, pores weaken the microstructure of the electrolyte resulting in lower fracture strength.

#### 3.2.1. Density of TiO_2_-Doped Na-β″-Alumina

**Figure 6:**
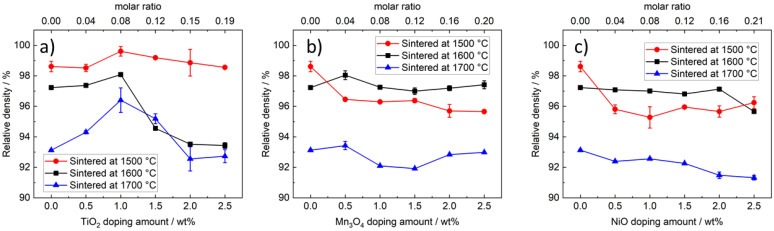
(**a**) Relative density of TiO_2_-doped Na-β″-alumina samples; (**b**) relative density of Mn_3_O_4_-doped Na-β″-alumina samples; (**c**) relative density of NiO-doped Na-β″-alumina samples.

Figure 6a displays the relative density of TiO_2_-doped Na-β″-alumina. The peak densities are achieved by a sintering temperature of 1500 °C and a doping amount of 1.0 wt% (99.6%/3.18 g cm^−3^). A temperature of 1600 °C leads to slightly lower densities with a maximum at 1 wt% doping (98.1%/3.14 g cm^−3^). Even higher doping amounts lead to a density drop. Similar behavior was found at sintering temperatures of 1700 °C. The maximum is reached at 1 wt% TiO_2_, but density reaches only 96.4%/3.09 g cm^−3^_._ Hence, 1 wt% of TiO_2_ doping leads to a density maximum regardless of the sintering temperature. Higher doping amounts and sintering temperatures lead to relatively lower densities. The higher density hints towards a liquid-assisted sintering process at a sintering temperature of 1500 °C. The porosity increased most likely in consequence of oversintering, caused by pore agglomeration, and increased sublimation of Na_2_O (sublimating point 1275 °C) [34].

#### 3.2.2. Density of Mn_3_O_4_- and NiO-Doped Na-β″-Alumina

Figure 6b shows the influence of Mn_3_O_4_ doping on the relative densities of Na-β″-alumina. Samples sintered at 1600 °C or 1700 °C show relative densities of about 97.5% and 93.0%, respectively, not depending on the doping amount. The density of samples sintered at 1500 °C drops from 98.6%/3.16 g cm^−3^ to 96.0%/3.11 g cm^−3^ due to 0.5 wt% of Mn_3_O_4_ doping.

The relative density of NiO-doped Na-β″-alumina (Figure 6c) has a minimal downward tendency after sintering at 1600 °C or 1700 °C. At sintering temperatures of 1500 °C, a similar trend to Mn_3_O_4_-doped samples can be observed; 0.5 wt% of NiO doping reduces the relative density from 98.6%/3.16 g cm^−3^ to 96.0%/3.11 g cm^−3^.

In summary, Mn_3_O_4_ and NiO doping shows a slight influence on the relative density at sintering temperatures of 1600 °C and 1700 °C, while the relative density is lowered at sintering temperatures of 1500 °C.

### 3.3. SEM and EDX Analysis

To analyze the influence of 3d transition metal doping and the sintering temperature on the microstructure, SEM studies were carried out. Subsequently, an EDX mapping was performed to investigate the dopant distribution within the microstructure.

#### 3.3.1. Influence of TiO_2_ Doping on the Microstructure

**Figure 7:**
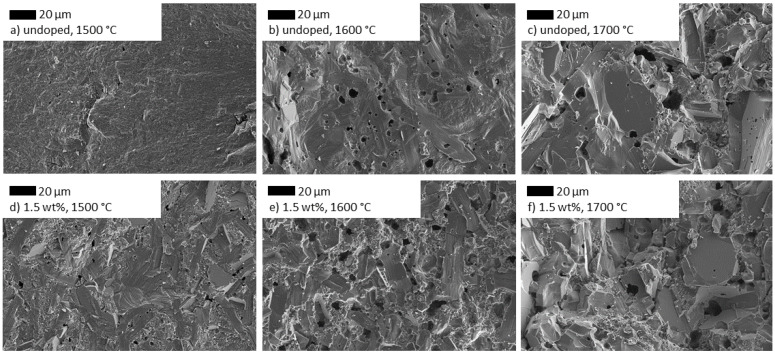
SEM images of Na-β″-alumina samples sintered at 1500 °C, 1600 °C, or 1700 °C; (**a**–**c**) undoped samples; (**d**–**f**) Na-β″-alumina doped with 1.5 wt% TiO_2_. Images (**a**,**b**,**d**,**e**) were rearranged and reprinted with permission from Dirksen et al. [13].

Figure 7 illustrates the impact of TiO_2_ doping on Na-β″-alumina samples for different sintering regimes. For undoped samples, a drastic microstructure changes from sintering temperatures of 1500 °C to 1700 °C is obvious (Figure 7a–c). The microstructure is characterized by fine grains (<10 µm) after sintering at 1500 °C, while higher temperatures result in extensive grain growth and pores with a diameter of up to 20 µm (Figure 7a–c). Figure 7d, e shows Na-β″-alumina doped with 1.5 wt% TiO_2_ sintered at 1500 °C, 1600 °C, or 1700 °C. The most apparent change originating from TiO_2_ doping was found for a sintering temperature of 1500 °C. In comparison to the undoped sample, large and strongly anisotropic, directional grown grains are present. Sintering temperatures above 1500 °C result in more pores. The increase is in good agreement with the observed decreased relative densities of samples sintered at higher temperatures.

**Figure 8:**
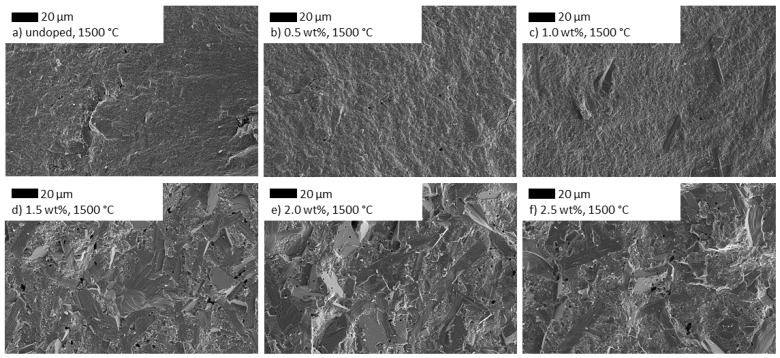
SEM images of Na-β″-alumina samples doped with different amounts of TiO_2_ (**b**–**f**) in comparison to an undoped sample (**a**). The images were rearranged and reprinted with permission from Dirksen et al. [13].

The influence of different TiO_2_-doping amounts at a constant sintering temperature on the microstructure is depicted in Figure 8. A doping amount of 0.5 wt% does not reveal any changes in comparison to an undoped sample, but 1.0 wt% of TiO_2_ doping results in occasional large crystals with a diameter of about 100 µm. Even higher doping amounts cause a microstructure dominated by large crystals but smaller pores than the samples sintered at 1600 °C.

**Figure 9:**
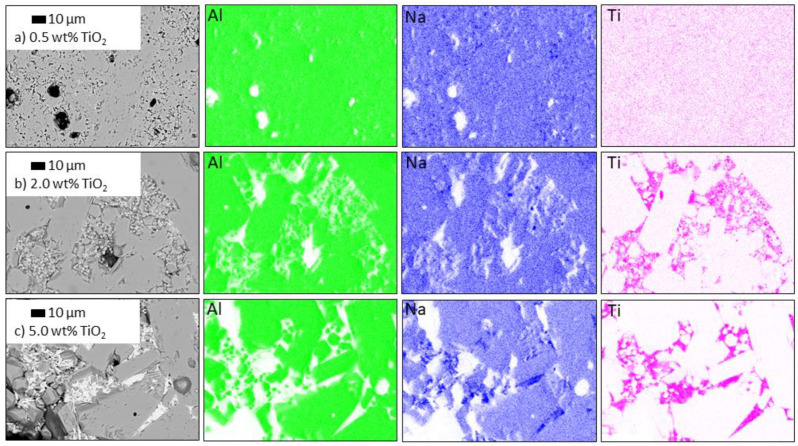
EDX mapping and backscattering SEM images of TiO_2_-doped, polished Na-β″-alumina samples (sintered at 1600 °C). (**a**) 0.0 wt% TiO_2_ (**b**) 2.0 wt% TiO_2_ (**c**) 5.0 wt% TiO_2_. Al is labeled green; Na is labeled blue; Ti is labeled pink.

**Figure 10:**
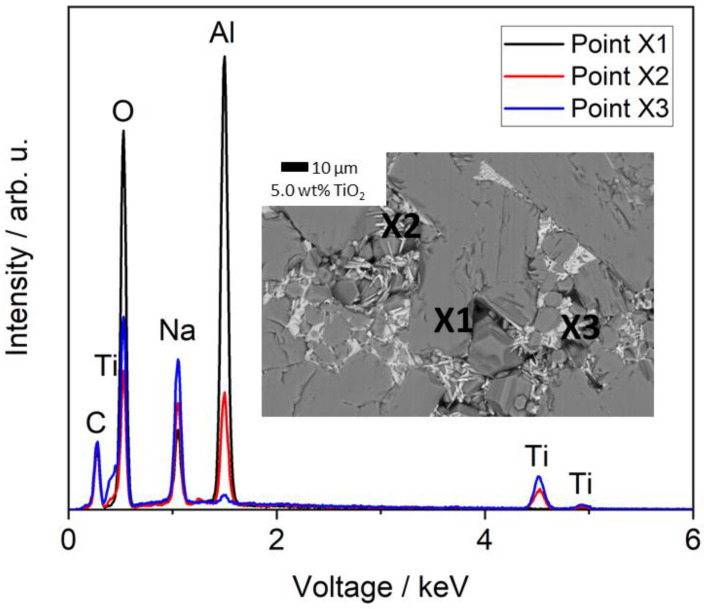
EDX point analysis of a Na-β″-alumina sample doped with 5.0 wt% TiO_2_ (sintered at 1600 °C). The measuring points are marked in the backscattering-SEM image.

To further investigate the influence of TiO_2_ doping on the microstructure of Na-β″-alumina, backscattering SEM, EDX mapping (Figure 9), and EDX spot analysis (Figure 10) were performed. Ti was not located by EDX mapping or an EDX spot analysis in the sample slightly doped with 0.5 wt% TiO_2_ (Figure 9a). The EDX mapping of the sample doped with 2.0 wt% revealed areas of fine grains (<10 µm), where Ti was detected, while large grains (>20 µm) were completely free of Ti. The sample doped with 5.0 wt% showed a clear phase separation: dark-appearing grains having a low mean atomic number originating potentially from phases comprising solely Na, Al, and O, and in-between light-appearing grains having a higher mean atomic number potentially originating from additionally Ti. The Al mapping reveals a decreasing Al content within the brighter phase. The Na content within the bright phase is uneven, and only some areas show an increased Na content (best visible in Figure 9c).

A subsequent spot analysis of the sample doped with 5.0 wt% TiO_2_ (Figure 10) confirmed a different chemical composition of the bright appearing sections. Point X2 (Na_3.4_Al_3.9_Ti_4.2_O_16_) gave a characteristic Al signal, while point X3 (Na_2.0_Al_0.1_Ti_3.0_O_7_) displays only signals of Ti, Na, and O. The small Al peak is most likely caused by the surrounding area of darker grains. It should be mentioned that areas with the composition of X3 are more often than those similar to X2. Point X1 (Na_1.63_Al_10.8_O_17_) is directly placed on a dark grain, which shows the typical composition of pure Na-β″-alumina but no Ti. The results strongly indicate that Ti-ions are not replacing Al-ions within the Na-β″-alumina crystal structure but are located in secondary phases. This postulate is supported by an unchanged lattice constant for Na-β″-alumina despite TiO_2_ doping. Furthermore, the strong influence of TiO_2_ doping on the microstructure and densification of Na-β″-alumina, which is shown in this and previous studies [18,21,23], indicates a liquid-assisted sintering process, caused by substances such as NaLiTi_3_O_7_ or Na_1.97_Al_1.82_Ti_6.15_O_16_ (melting point: 1300 °C [35]). The chemical composition measured by EDX does not exactly fit the phases identified in the XRD patterns, which hints toward a non-stoichiometric mix of compounds. Furthermore, Li cannot be detected via EDX due to its small atomic number.

#### 3.3.2. Influence of Mn_3_O_4_ Doping on the Microstructure

**Figure 11:**
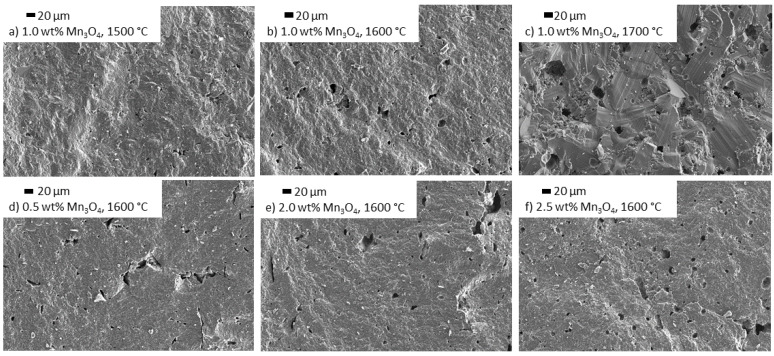
SEM images of Na-β″-alumina samples sintered at 1500 °C, 1600 °C, or 1700 °C, doped with different amounts of Mn_3_O_4_ (**b**–**f**) in comparison to an undoped sample (**a**).

**Figure 12:**
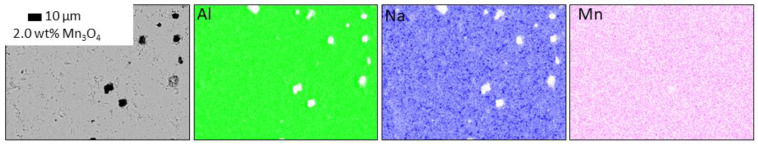
EDX mapping and backscattering SEM image of Mn_3_O_4_-doped, polished Na-β″-alumina samples (sintered at 1600 °C). Al is labeled green; Na is labeled blue; Mn is labeled pink.

The influence of Mn_3_O_4_ doping on Na-β″-alumina strongly differs from TiO_2_ doping. The microstructure of samples doped with 1.0 wt% Mn_3_O_4_ and sintered at 1500 °C or 1600 °C predominantly consist of small grains, while sintering at 1700 °C results in excessive grain growth. Higher doping amounts do not promote the formation of large grains (Figure 11a,d–f) at temperatures of 1600 °C. Since undoped Na-β″-alumina started excessive grain growth at 1600 °C, Mn_3_O_4_ suppresses the grain growth of Na-β″-alumina. The EDX mapping of a Mn_3_O_4_-doped sample, displayed in Figure 12, confirms an even distribution of Mn.

#### 3.3.3. Influence of NiO Doping on the Microstructure

**Figure 13:**
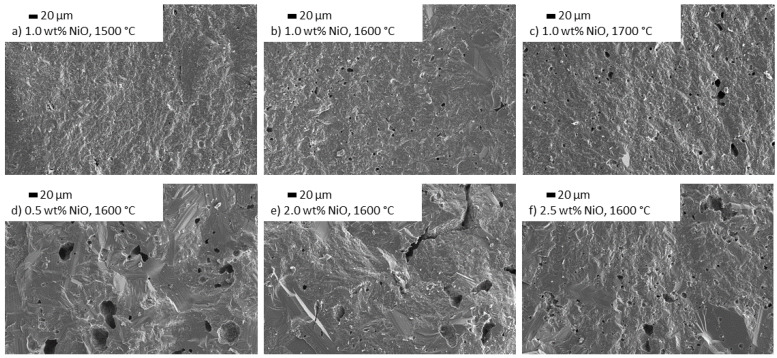
SEM images of Na-β″-alumina samples sintered at 1500 °C, 1600 °C, or 1700 °C, doped with different amounts of NiO (**b**–**f**) in comparison to an undoped sample (**a**).

**Figure 14:**
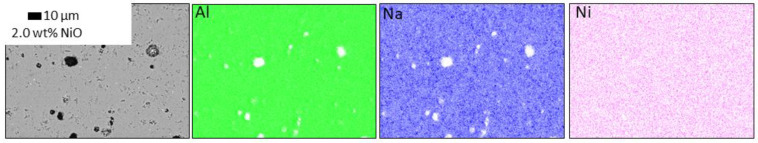
EDX mapping and backscattering SEM image of NiO-doped, polished Na-β″-alumina samples (sintered at 1600 °C). Al is labeled green; Na is labeled blue; Ni is labeled pink.

1.0 wt% NiO doped Na-β″-alumina shows a microstructure with fine grains and only occasional large grains for all tested sintering temperatures (Figure 13a–c). The variation of the NiO doping amount at a fixed sintering temperature of 1600 °C (Figure 13a,d–f) shows that 0.5 wt% of doping does not prevent excess grain growth. Samples doped with 2.0 wt% and 2.5 wt% show a fine-grained microstructure with a few scattered large grains. The EDX mapping (Figure 14) shows an even distribution of Ni over the whole sample. The isolated white dots visible in the backscattering electron SEM are caused by ZrO_2_ particles, which remained from the milling process.

Compared to this work, Zhu et al. [20] found different effects for Na-β″-alumina electrolytes doped with NiO. They observed increased densification of doped samples, which could not be confirmed. Most certainly, those differences can be explained by the different manufacturing processes. Zhu et al. added the dopant before calcining, while this work added the dopant after calcining. Moreover, Zhu et al. sintered their samples as low as 1400 °C for 2 h instead of choosing higher temperatures for a time of 0.5 h.

It can be stated that the addition of Mn_3_O_4_ and NiO suppresses the formation of large grains. Neither Mn_3_O_4_ nor NiO doping caused any formation of detectable secondary phases in XRD measurements, while the lattice parameters changed. This indicates incorporation of Mn and Ni into the crystal lattice of Na-β″-alumina. Furthermore, EDX measurements and backscattering SEM did not reveal any secondary phases related to Mn_3_O_4_/NiO doping. Therefore, it is likely, that Mn- and Ni-ions replace Al-ions within the spinel block as assumed by previous researchers [25,36].

### 3.4. Fracture Strength Analysis

**Figure 15:**
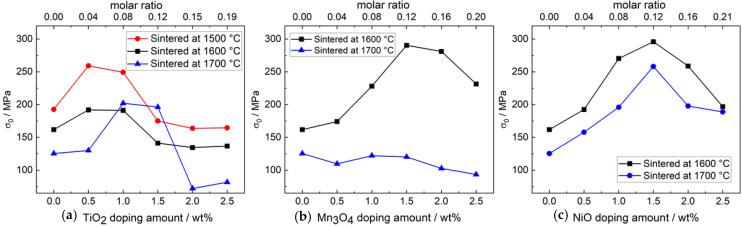
(**a**) Characteristic fracture strength σ_0_ of TiO_2_-doped Na-β″-alumina. The results of TiO_2_-doped samples and sintering temperatures of 1500 °C and 1600 °C are reprinted with permission from Dirksen et al. [13]. (**b**) Characteristic fracture strength σ_0_ of Mn_3_O_4_-doped Na-β″-alumina. (**c**) Characteristic fracture strength σ_0_ of NiO-doped Na-β″-alumina.

The fracture strength is an essential material property of Na-β″-alumina electrolytes. It limits the dimensions of an electrolyte that are required to withstanding the mechanical stresses occurring in an operating electrochemical cell. Figure 15 shows comparatively the characteristic fracture strengths σ_0_ for a failure probability of 63.2%. Tables S1–S3 present the exact values and additionally the distribution parameter m from data evaluation according to Weibull statistics.

#### 3.4.1. Influence of TiO_2_ Doping on the Fracture Strength

For undoped samples, a clear decrease of characteristic fracture strength was found for increasing sintering temperatures. The σ_0_ value of samples sintered at 1500 °C was 193 MPa, while sintering at 1700 °C decreased σ_0_ to 125 MPa. The reason for this decrease in fracture strength can be found in the increased porosity and grain size with rising sintering temperature.

Small amounts of TiO_2_ doping increase the characteristic fracture strength of Na-β″-alumina electrolytes regardless of the sintering temperature. Doping of 0.5 wt% TiO_2_ and a sintering temperature of 1500 °C led to the maximum characteristic fracture strength of 259 MPa. Higher doping amounts than 1.0 wt% TiO_2_ causes an abrupt decrease of the characteristic fracture strength. This observation corresponds well with the SEM images, which prove extensive grain growth. At higher sintering temperatures, the fracture strength σ_0_ follows the same trend as the relative density. It indicates that porosity becomes the most impactful factor. Chen et al. [24] found a maximum fracture strength of 230 MPa at a similar doping amount of 0.5 wt% TiO_2_.

#### 3.4.2. Influence of Mn_3_O_4_ Doping on the Fracture Strength

Compared to TiO_2_- and NiO-doped samples, the distribution of fracture strength of Mn_3_O_4-_doped samples appears different. At sintering temperatures of 1600 °C, the characteristic fracture strength increases from 162 MPa to 290 MPa by doping with 1.5 wt% Mn_3_O_4_. At a sintering temperature of 1700 °C, no further upward trend is observed. Those results correspond well with the SEM images in the section “3.3 SEM and EDX analysis”. While Mn_3_O_4_ doping was able to suppress extensive grain growth at 1600 °C, temperatures of 1700 °C resulted in excessive grain growth.

#### 3.4.3. Influence of NiO Doping on the Fracture Strength

NiO is the most efficient dopant in terms of increasing fracture strength. Doping with 1.5 wt% NiO increased the characteristic fracture strength from 162 MPa to 296 MPa after sintering at 1600 °C, because it prevents extensive grain growth. The impact of Ni-ion incorporation into the crystal lattice on the fracture strength is not clear yet. However, it seems likely that very high doping amounts can destabilize the crystal lattice. Zhu et al. [20] also doped Li-stabilized Na-β″-alumina with NiO and observed the same fracture strength maximum at 296 MPa, but at a doping amount of 0.25 wt%. A reason for the different results might be the usage of different reactants. Lu et al. used α-Al_2_O_3_ and Na_2_C_2_O_4_, while AlO(OH) and Na_2_CO_3_ were used in this work. Especially the Al source influences the Na-β″-alumina ceramic´s fracture strength [14,37].

### 3.5. Ionic Conductivity Analysis

High ionic conductivity of the electrolyte is crucial for a highly efficient cell system. Several factors are influential for the ionic conductivity of polycrystalline Na-β″-alumina electrolytes. First, grain size and extensive grain growth play an important role [13]. Large grains decrease the grain boundary resistivity since fewer grain boundaries have to be passed. Additionally, large, anisotropic, and 2D-conductive grains lower the tortuosity and therefore shorten the ion pathway, which decreases the effective resistance [38]. The severity of anisotropy was visible in the intensity of the reflexes 006. Second, the porosity and the related density of the solid electrolyte influence ionic conductivity. An increasing porosity extends the pathway for the sodium ions, and the ionic conductivity decreases. Third, the intrinsic conductivity of the Na-β″-alumina grains possibly changed due to the incorporation of foreign ions into the lattice can have an impact on the ionic conductivity. Fourth, the phase content of the highly conductive Na-β″-alumina within the electrolyte is of great importance. Due to the complex phase formation process of poly crystalline Na-β″-alumina, a mixture of ß” and ß-alumina as well as some secondary phases will always be present in the electrolyte. Accordingly, the Na-β″-alumina content and the sensitive process for electrolyte production needs to be optimized. Figure S1 shows representative Nyquist plots from impedance spectroscopy measurements and the corresponding fits used to determine R_b_ and R_gb_.

#### 3.5.1. Influence of TiO_2_ Doping on the Ionic Conductivity

**Figure 16:**
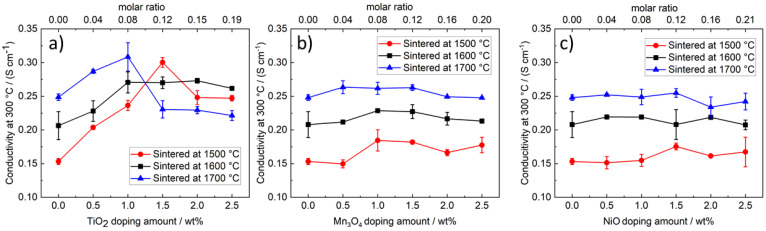
Ionic conductivity at 300 °C of differently doped and sintered Na-β″-alumina samples. (**a**) TiO_2_-doped Na-β″-alumina; the results of TiO_2_-doped samples sintered at temperatures of 1500 °C and 1600 °C are reprinted with permission from Dirksen et al. [13] (**b**) Mn_3_O_4_-doped Na -β″-alumina; (**c**) NiO-doped Na -β″-alumina.

Figure 16 displays the overall ionic conductivity at 300 °C of the different samples. For undoped electrolyte samples, the conductivity increases from 0.15 S cm^−1^ over 0.21 S cm^−1^ to 0.25 S cm^−1^ with an increasing sintering temperature from 1500 °C over 1600 °C to 1700 °C. This increase can be addressed to the increasing grain size since the phase content of the sample stays nearly constant (see XRD results in Section 3.1). The porosity, which increases due to the higher sintering temperature (Figure 6), is not high enough to counter the effect of excessive grain growth. The grain growth is also causal for the decrease of characteristic fracture strength discussed in Section 3.4.

The ionic conductivity of the electrolyte samples doped with TiO_2_ is displayed in Figure 16a. Next to the sintering temperature, the TiO_2_ doping amount influences the measured conductivity enormously. At sintering temperatures of 1500 °C, the ionic conductivity is increased from 0.15 S cm^−1^ to 0.30 S cm^−1^ by adding 1.5 wt% TiO_2_. SEM images show that in between 1.0 wt% and 1.5 wt% of dopant and at a sintering temperature of 1500 °C, the excessive grain growth takes place. The grain growth leads to an increase in conductivity while the fracture strength decreases. A further increase of the TiO_2_ doping amount at 1500 °C results in a decreasing conductivity. The formation of pores and the corresponding decrease of material density (Figure 6) obviously compensates for the positive effect of grain growth. The conductivity is lowered from approximately 0.30 S cm^−1^ to 0.25 S cm^−1^.

Independently from the doping level, at sintering temperatures of 1600 °C and 1700 °C, an excessive grain growth is present, which is mirrored in high ionic conductivities.

TiO_2_ doping with 1.0 wt% results in the highest conductivities and the highest material densities.

The influence of the Na-β″-alumina phase content on the ionic conductivity is low since the phase content changes only marginally at different temperatures and doping amounts (Table 1). Ti-ions are not incorporated into the Na-β″-alumina crystal lattice; therefore, a change of the intrinsic conductivity of Na-β″-alumina is not expected. Na-ion depletion of the Na-β″-alumina phase due to the formation of Na and Ti secondary phases might have a minimal influence. However, a massive depletion seems unlikely because all tested samples contained a small yet notable amount of Na-rich NaAlO_2_.

**Figure 17:**
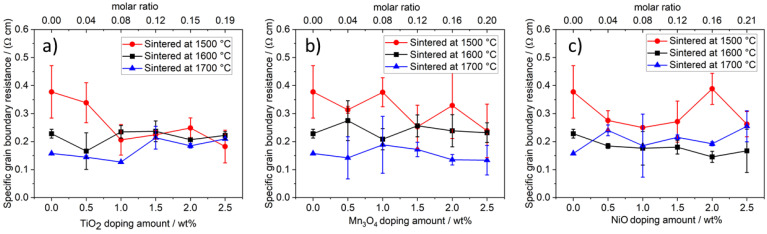
Specific grain boundary resistance of Na-β″-alumina samples. (**a**) TiO_2_-doped Na-β″-alumina; (**b**) Mn_3_O_4_-doped Na-β″-alumina; (**c**) NiO-doped Na-β″-alumina.

The specific grain boundary resistances R_sgb_ (see Figure 17a) are in good agreement with the observed microstructure: The specific grain boundary resistance of undoped samples, sintered at 1500 °C, shrinks from 0.38 Ω cm to 0.16 Ω cm for samples sintered at 1700 °C. All samples with a microstructure shaped by large grains have a specific grain boundary resistance of less than 0.25 Ω cm. Samples with finer grains (0.0 and 0.5 wt% TiO_2_ doping/sintered at 1500 °C) show resistance of 0.38 Ω cm and 0.34 Ω cm because Na-ions must cross more grain boundaries. Potentially, impedance spectroscopy can be used to get an impression of the microstructure of Na-β″-alumina electrolytes. It is already done for other materials [39,40].

#### 3.5.2. Influence of Mn_3_O_4_ and NiO Doping on the Ionic Conductivity

The influence of Mn_3_O_4_ and NiO doping on the overall ionic conductivity (Figure 16) of Na-β″-alumina electrolytes was much lower compared to that of TiO_2_. Only a negligible impact was detected. This observation is remarkable because the grain size and thereby the characteristic fracture strength was lowered by those dopants. Therefore, lower conductivities should be expected. Furthermore, the relative density of the Na-β″-alumina samples was only slightly influenced by doping. Hence, it seems likely that the intrinsic conductivity of the Na-β″-alumina crystals changes due to the incorporation of Mn- or Ni-ions. Boilot et al. [36] assume that M^2+^ ions like Mn^2+^ and Ni^2+^, which replace Al^3+^, can reduce the number of interstitial oxygen ions because fewer oxygen ions are needed to achieve electric neutrality. The reduced number of interstitial oxygen ions is considered to enhance the diffusion of Na-ions within the ion conductive plane.

The specific grain boundary resistance of Mn_3_O_4_-doped samples (Figure 17b) decreases with higher sintering temperatures, while the doping amount shows only little influence. NiO-doped samples behave similarly but show faintly lower grain boundary resistance at a sintering temperature of 1600 °C.

## 4. Conclusions

Li-stabilized Na-β″-alumina powder was doped with three different 3d transition metal oxides (TiO_2_, Mn_3_O_4_, and NiO), granulated, pressed, and sintered at different temperatures. For the first time, the mechanism from TiO_2_ doping of Li-stabilized Na-β″-alumina was clarified and understood. It was found that TiO_2_ doping promotes the formation of secondary phases such as NaLiTi_3_O_7_ and Na_1.97_Al_1.82_Ti_6.15_O_16_. This assumption was supported by results from SEM–EDX measurements, which clearly proved the absence of Ti inside the Na-β″-alumina grains. Since the melting point of Na_2_Al_2_Ti_6_O_16_ amounts to 1300 °C [35], liquid-assisted sintering can be assumed. This assumption was confirmed by the successful synthesis of highly conductive electrolytes (0.30 S cm^−1^ at 300 °C) and the formation of a coarse-grained microstructure even at a very low sintering temperature of only 1500 °C. It is not clear yet to what extent the secondary phases can influence the long-term stability of Na-β″-alumina regarding electrochemical stability and dendrites formation.

For Mn_3_O_4_- and NiO-doped Na-β″-alumina, no formation of secondary phases and according liquid-assisted sintering was observed. Only minor changes in the ionic conductivity were measured independently from the doping level. SEM–EDX and XRD measurements indicated that Mn- and Ni-ions occupy the Al-positions in the lattice of the Na-β″-alumina crystals, while the Ti-ions are located in secondary phases.

All three dopants increased the characteristic fracture strength of the electrolytes. The enhancement was assigned to changes in the microstructure. The addition of 1.5 wt% NiO increased the characteristic fracture strength from 162 to 296 MPa (at sintering temperature of 1600 °C).

From the comparative studies, it can be summarized that TiO_2_ is the dopant that affects the Na-β″-alumina properties most intensively. It has the potential to reduce the sintering temperature, increase fracture strength, and enhance ionic conductivity. A good balance of those parameters was found at a doping amount of 1.0 wt% TiO_2_ and a sintering temperature of 1500 °C. The low sintering temperature decreases the energy consumption of the manufacturing process. Simultaneously, the characteristic fracture strength was increased from 193 to 249 MPa. The ionic conductivity at 300 °C amounts to 0.22 S cm^−1^ for this material.

The reported results suggest that 3d transition metal doping represent an effective method to adjust the electrolyte properties on the one hand and optimize the energy consumption for electrolyte production on the other hand. By targeted doping, the production of thin-walled, stable, and highly conductive Na-β″-alumina electrolytes for Na-based batteries becomes feasible. Future research will address the mixture of different dopants such as TiO_2_ and NiO to evaluate if an even higher characteristic fracture strength can be achieved at low sintering temperatures. Furthermore, tests on Na/NiCl_2_-cells in order to test the long-term stability of doped Na-β″-alumina electrolytes are pending.

## Figures and Tables

**Table 1:** Na-β″-alumina phase content [wt%] of differently sintered and doped samples.

	TiO_2_ Doped			Mn_3_O_4_ Doped		NiO Doped	
Doping Amount/wt%	1500 °C	1600 °C	1700 °C	1600 °C	1700 °C	1600 °C	1700 °C
0	96.4 ± 0.1	96.3 ± 0.5	95.5 ± 1.7	96.3 ± 0.5	95.5 ± 1.7	96.3 ± 0.5	95.5 ± 1.7
0.5	96.4 ± 0.4	96.6 ± 0.4	96.2 ± 0.3	94.9 ± 0.3	93.8 ± 3.6	97.0 ± 0.1	94.6 ± 0.5
1.0	96.8 ± 0.5	96.4 ± 0.4	98.0 ± 0.4	94.1 ± 4.1	94.4 ± 1.0	96.3 ± 0.4	95.2 ± 0.5
1.5	95.5 ± 0.5	94.0 ± 0.6	94.1 ± 1.0	95.7 ± 0.5	95.3 ± 1.0	96.7 ± 1.5	96.7 ± 0.2
2.0	96.3 ± 0.8	95.9 ± 0.5	90.8 ± 0.6	93.5 ± 0.3	95.6 ± 2.4	94.8 ± 0.2	97.1 ± 0.5
2.5	94.8 ± 0.5	95.3 ± 0.2	96.7 ± 0.8	95.2 ± 0.2	93.9 ± 2.1	94.4 ± 0.2	96.8 ± 0.9
5.0	96.6 ± 1.1	92.5 ± 0.3	94.2 ± 1.7	93.9 ± 1.6	93.2 ± 0.9	95.5 ± 0.3	95.6 ± 1.2

## Data Availability

The data presented in this study are available on request from the corresponding author.

## Supplementary Materials

The following are available online at https://www.mdpi.com/article/10.3390/ma14185389/s1, Figure S1: Nyquist plots of differently doped Na-β″-alumina samples recorded at 300 °C. (a) 1.0 wt% TiO_2_ doped (b) 1.0 wt% Mn_3_O_4_ doped (c) 1.0 wt% NiO doped.; Table S1: Characteristic fracture σ_0_ and distribution parameter m of TiO_2_ doped Na-β″-alumina sintered at temperatures from 1500 °C to 1700 °C; Table S2: Characteristic fracture σ_0_ and distribution parameter m of Mn_3_O_4_ doped Na-β″-alumina sintered at temperatures 1600 °C respectively 1700 °C; Table S3: Characteristic fracture σ_0_ and distribution parameter m of NiO doped Na-β″-alumina sintered at temperatures 1600 °C respectively 1700 °C
