# Supplementary material for: Influence of 3d Transition Metal Doping on Lithium Stabilized Na-β″-Alumina Solid Electrolytes
Source: Materials (Basel). 2021 Sep 17;14(18):5389. doi: 10.3390/ma14185389 (PMC8466469; doi:10.3390/ma14185389)
Supplement: Supplementary file 1 [file materials-14-05389-s001.zip › materials-1376044-supplementary.pdf]

materials

MDPI

Supplementary Materials

Influence of 3d Transition Metal Doping on Lithium Stabilized Na-β''-alumina Solid Electrolytes

Cornelius L. Dirksen *, Karl Skadell, Matthias Schulz, Micha P. Fertig and Michael Stelter

Fraunhofer Institute for Ceramic Technologies and Systems IKTS, Michael-Faraday-Str. 1, 07629 Hermsdorf, Germany; Cornelius.Dirksen@ikts.fraunhofer.de (C.D.); Karl.Skadell@ikts.fraunhofer.de (K.S.); Matthias.Schulz@ikts.fraunhofer.de (M.Sc.); Micha.Philip.Fertig@ikts.fraunhofer.de (M.P.); Michael.Stelter@ikts.fraunhofer.de (M.S.)
* Correspondence: Cornelius.Dirksen@ikts.fraunhofer.de

Table S1. Characteristic fracture σ_0 and distribution parameter m of TiO_2 doped Na-β''-alumina sintered at temperatures from 1500 °C to 1700 °C.

TiO_2 doping amount / wt%	σ_0 (Sintered at 1500 °C) / MPa	m	σ_0 (Sintered at 1600 °C) / MPa	m	σ_0 (Sintered at 1700 °C) / MPa	m
0.0	193	13	162	4.2	125	5.5
0.5	259	9.7	192	5.7	130	12
1.0	249	5.1	191	9.3	202	8.4
1.5	175	14	141	12	196	7.1
2.0	164	12	134	17	72.0	3.3
2.5	165	20	137	16	81.6	5.0

Table S2. Characteristic fracture σ_0 and distribution parameter m of Mn_3O_4 doped Na-β″-alumina sintered at temperatures 1600 °C respectively 1700 °C.

Mn_3O_4 doping amount / wt%	σ_0 (Sintered at 1600 °C) / MPa	m	σ_0 (Sintered at 1700 °C) / MPa	m
0.0	162	4.2	125	5.5
0.5	174	8.6	110	11
1.0	228	8.8	122	13
1.5	290	10	120	7.4
2.0	281	12	103	5.8
2.5	231	15	93.4	10

Table S3. Characteristic fracture σ_0 and distribution parameter m of NiO doped Na-β″-alumina sintered at temperatures 1600 °C respectively 1700 °C.

NiO doping amount / wt%	σ_0 (Sintered at 1600 °C) / MPa	m	σ_0 (Sintered at 1700 °C) / MPa	m
0.0	162	4.2	125	5.5
0.5	193	10	158	5.8
1.0	270	4.4	196	6.6
1.5	296	6.1	258	6.1
2.0	259	7.0	198	18
2.5	197	4.5	189	6.3

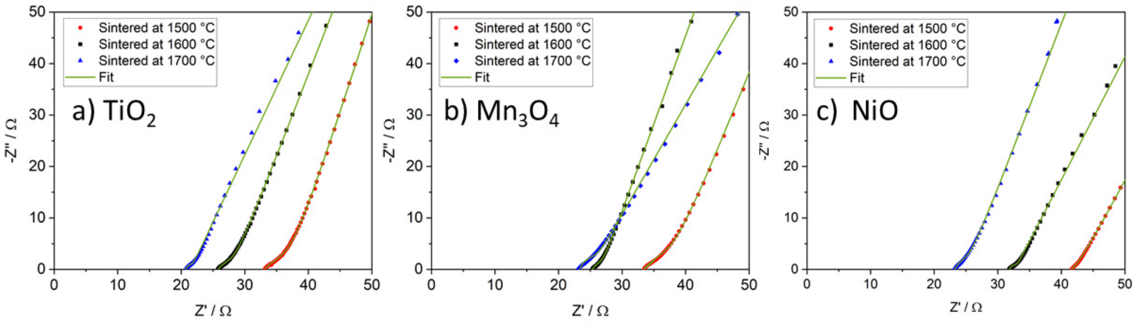

Figure S1. Nyquist plots of differently doped Na-β″-alumina samples recorded at 300 °C. (**a**) 1.0 wt% TiO_2 doped (**b**) 1.0 wt% Mn_3O_4 doped (**c**) 1.0 wt% NiO doped.
